# Noninvasive, *In Vivo* Assessment of Mouse Retinal Structure Using Optical Coherence Tomography

**DOI:** 10.1371/journal.pone.0007507

**Published:** 2009-10-19

**Authors:** M. Dominik Fischer, Gesine Huber, Susanne C. Beck, Naoyuki Tanimoto, Regine Muehlfriedel, Edda Fahl, Christian Grimm, Andreas Wenzel, Charlotte E. Remé, Serge A. van de Pavert, Jan Wijnholds, Marek Pacal, Rod Bremner, Mathias W. Seeliger

**Affiliations:** 1 Division of Ocular Neurodegeneration, Centre for Ophthalmology, Institute for Ophthalmic Research, University of Tuebingen, Tuebingen, Germany; 2 Institute of Animal Welfare, Ethology and Animal Hygiene, Faculty of Veterinary Medicine, Ludwig-Maximilians-University, Munich, Germany; 3 Laboratory of Retinal Cell Biology, University of Zurich, Zurich, Switzerland; 4 Neuromedical Genetics, Netherlands Institute for Neuroscience, Amsterdam, The Netherlands; 5 Toronto Western Research Institute, University Health Network, Departments of Ophthalmology and Visual Science, and Laboratory Medicine and Pathobiology, University of Toronto, Toronto, Ontario, Canada; Vrije Universiteit Amsterdam, Netherlands

## Abstract

**Background:**

Optical coherence tomography (OCT) is a novel method of retinal *in vivo* imaging. In this study, we assessed the potential of OCT to yield histology-analogue sections in mouse models of retinal degeneration.

**Methodology/Principal Findings:**

We achieved to adapt a commercial 3^rd^ generation OCT system to obtain and quantify high-resolution morphological sections of the mouse retina which so far required *in vitro* histology. OCT and histology were compared in models with developmental defects, light damage, and inherited retinal degenerations. In conditional knockout mice deficient in retinal retinoblastoma protein Rb, the gradient of *Cre* expression from center to periphery, leading to a gradual reduction of retinal thickness, was clearly visible and well topographically quantifiable. In *Nrl* knockout mice, the layer involvement in the formation of rosette-like structures was similarly clear as in histology. OCT examination of focal light damage, well demarcated by the autofluorescence pattern, revealed a practically complete loss of photoreceptors with preservation of inner retinal layers, but also more subtle changes like edema formation. In *Crb1* knockout mice (a model for Leber's congenital amaurosis), retinal vessels slipping through the outer nuclear layer towards the retinal pigment epithelium (RPE) due to the lack of adhesion in the subapical region of the photoreceptor inner segments could be well identified.

**Conclusions/Significance:**

We found that with the OCT we were able to detect and analyze a wide range of mouse retinal pathology, and the results compared well to histological sections. In addition, the technique allows to follow individual animals over time, thereby reducing the numbers of study animals needed, and to assess dynamic processes like edema formation. The results clearly indicate that OCT has the potential to revolutionize the future design of respective short- and long-term studies, as well as the preclinical assessment of therapeutic strategies.

## Introduction

Mice are important model organisms in many areas of science. The retina as part of the brain offers the unique opportunity to directly visualize changes associated with neurodegenerative disorders and vascular alterations [1], or during the preclinical assessment of pharmacological approaches [2]. However, the current need to sacrifice animals for histological examinations at different time points interferes with the ability to follow the disease process and to monitor therapeutic or side effects over time in the same individuals.

**Figure 1:**
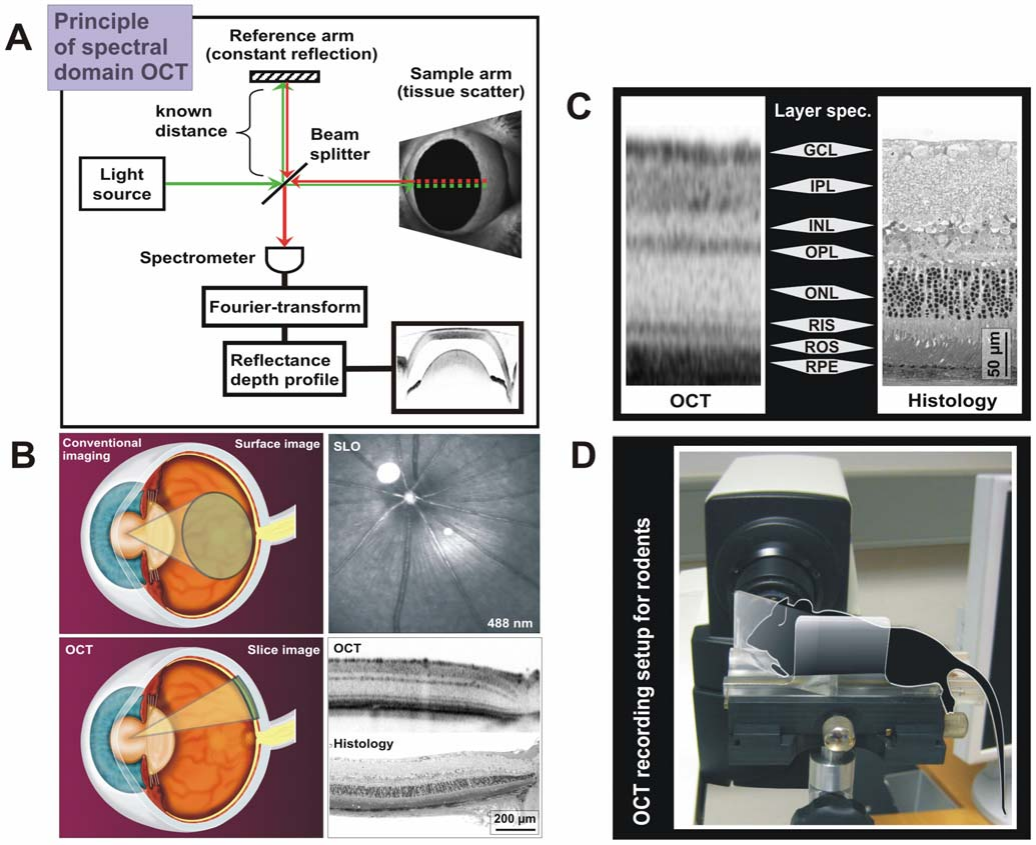
Principle of Optical Coherence Tomography (OCT) and its application in rodents. A) Schematic diagram of spectral-domain (SD) OCT. Green arrows indicate efferent, red arrows afferent light. B) Comparison of OCT to conventional ophthalmic imaging techniques. Top left: Conventional techniques either yield surface images of the retina (“fundus”), or in case of Scanning-Laser Ophthalmoscopy (SLO), confocal horizontal sections. Top right: Example of an SLO image of the central murine retina. Bottom left: In contrast, the OCT provides high-resolution vertical sections. Bottom right: Example of an OCT slice from the central murine retina in comparison to matching standard light microscopy. C) Representation of retinal layers in OCT and histology. See text for details. D) OCT recording setup for rodents. The schematic drawing of a mouse marks the recording position on the XYZ table. The eye is directly facing the OCT recording unit with a 78 dpt. lens attached.

Optical coherence tomography (OCT) is a novel method of retinal imaging [3]. Technically, OCT uses a weakly coherent infrared laser to analyze the reflectance properties of a sample [3]. The spectral domain mode (Fig. 1 A) currently outmatches the time domain mode as it combines high resolution with high recording speed. Each resulting data set consists of a large number of topographically ordered depth profiles (A-Scans), which together represent a two-dimensional slice (similar to an ultrasound scan) across the sample. Several consecutive slices together may be used to generate a three-dimensional data set (“volume scan”).

State-of-the-art imaging in rodents based on conventional fundus cameras or even confocal scanning-laser ophthalmoscopy (SLO) provides predominantly surface information but has a very restricted depth resolution [1] (Fig. 1B). This has been a severe limitation in animal studies. The OCT, in contrast, provides a high-resolution depth profile based on reflectivity that correlates well with histomorphological sections (Fig. 1C). However, the resulting image is different as conventional imaging and histology are based on absorption (light shade - little absorption, dark shade - strong absorption), whereas OCT is based on reflectivity (light shade - weak reflectivity, dark shade - strong reflectivity). In particular, membranous surfaces are extremely well detected in OCT regardless of their physical extension. Consequently, membrane-rich but less optically dense layers like plexiform and nerve-fiber layers are represented in a darker shade of gray than optically more dense layers with less membrane content like the outer nuclear layer (Fig. 1C). It is thus that the OCT provides not only refined depth resolution but also entirely new information about the sample structure.

Recent studies with custom-made equipment support the applicability of OCT in rodents, the ability to detect retinal lesions, and the potential to follow disease processes over time [4], [5]. In this work, we adapted a high-resolution 3^rd^ generation OCT system (Heidelberg Engineering Spectralis™) for use in small animal models to obtain detailed histology-analogue sections of the retina *in vivo*, without the need for an internal modification of the OCT device. This approach is superior to the use of custom-made experimental setups in terms of availability, reproducibility, familiarization, and standardization, and will thus be valuable for the future spread of this technique among the scientific community. The setup with an animal in place is shown in Fig. 1D and further described in the methods section.

## Results

Following an initial comparison between OCT and histology in C57/BL6 wild-type mice, we have studied the representation of the phenotype in models with developmental defects (*Rb* gene), light damage, and inherited retinal degenerations (*Nrl* & *Crb1*genes).

### Comparison of OCT and histology

**Figure 2:**
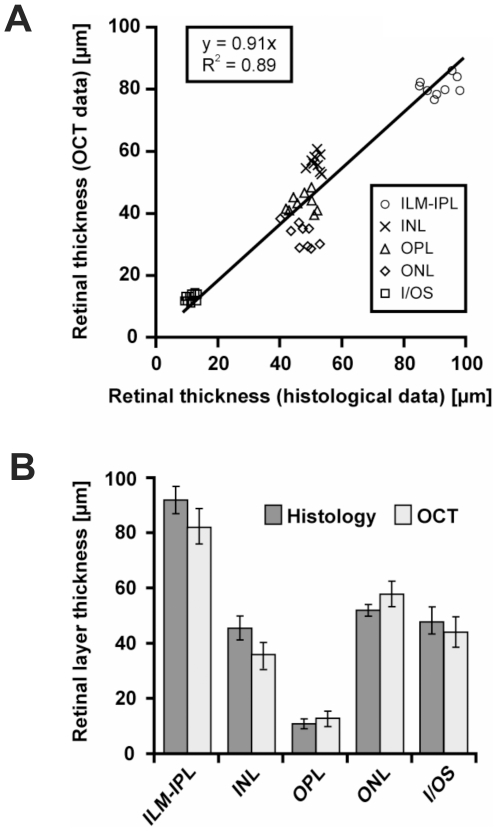
OCT and histological morphometric data in rodents. Relationship between OCT and histological morphometry in C57/BL6 mice broken down to retinal layers. A) Correlation of histological and OCT data. Pearson's correlation coefficient (R^2^) is based on the data of all quantified retinal layers. B) Comparison of retinal layer thickness between histology (dark) and OCT (light). There was no statistically significant difference between histological analysis and OCT-based quantification in any retinal layer using Student's t-test at a significance level of p<0.05. All data are reported as mean values±standard deviation (error bars).

The correspondence of layers in morphological OCT and histological sections (qualitatively shown in Fig. 1 D), was examined quantitatively in C57/BL6 wild-type mice (Fig. 2). No significant difference in layer thickness was detected between the two methods (Student's t-test at a significance level of p<0.05), and the best linear fit of the data yielded a correlation coefficient R^2^ of 0.89 (Fig. 2 A). Due to differences in tissue processing techniques, the slope of the fit may vary between labs; it is thus important to obtain a set of local control data for own studies. The average dimensions of the individual layers are given in Fig. 2B, together with their respective standard deviations, further confirming the close agreement between histology and OCT.

### Light damage

**Figure 3:**
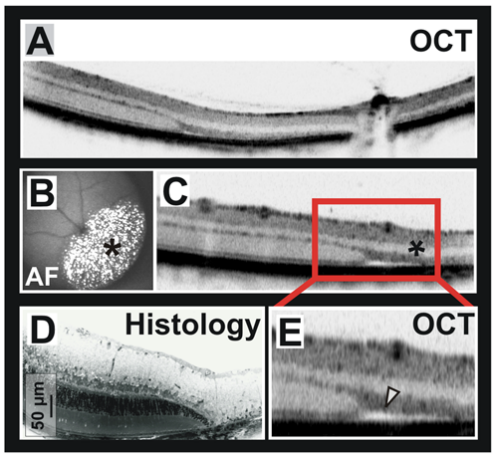
OCT assessment of light-induced murine retinal degeneration. A) OCT section across the central retina, containing adjacent damaged and non-damaged areas. B) Demarcation of the damaged area *in vivo* by SLO autofluorescence (AF) imaging based on fluorescent photoreceptor debris (marked by an asterisk). C) Detail of the transition zone between damaged and non-damaged retina in a). The asterisk marks the damaged area as in B). D), E) Comparison of the representation of light-induced retinal damage in histology and OCT. The arrowhead in the OCT image points towards a site of retinal edema.

Photoreceptor apoptosis is a common final path of many inherited and induced retinal neurodegenerations. Typically, the outer retina mainly formed by photoreceptor cell bodies and their inner and outer segments degenerates and subsequently vanishes without major alterations of the inner retinal layers. Whereas inherited diseases commonly affect the retina gradually, i.e. the course of a degeneration takes some time and its topographical distribution may not be uniform, focal light exposure (particularly high intensity blue light) produces strictly localized lesions adjacent to practically normal retina within a short period of time [6], allowing a direct comparison between damaged and non-damaged areas (Figure 3 A). The huge amount of autofluorescent, lipid-rich debris from photoreceptor outer segments that cannot be processed by the retinal pigment epithelium and glial cells clearly demarcates the area of damage in SLO autofluorescence imaging (Fig. 3 B). The selective loss of the outer retina in the exposed region (marked with an asterisk) is very prominent (Fig. 3 C). Also, the transition zone (Fig. 3 D,E) closely matches that in a respective histomorphological section, but preserves the capability to follow the development of such degenerative changes over time, an important added value of *in vivo* diagnostics. A novel finding in rodents was the discovery of a site of edema formation (arrowhead in Fig. 3 E), a pathological alteration that does not endure the tissue processing required for *in vitro* methods, and is thus not visible in traditional histology. Edema was present in all (4/4) of the treated cases 3 days following the exposure, and was still present one week later (4/4). In all cases, it formed a ring around the light damage area. Edema is a common finding in human retinal diseases. Its appearance in OCT has been extensively studied, and these findings closely resemble the situation in our data [7].

### *Nrl* knockout mouse

**Figure 4:**
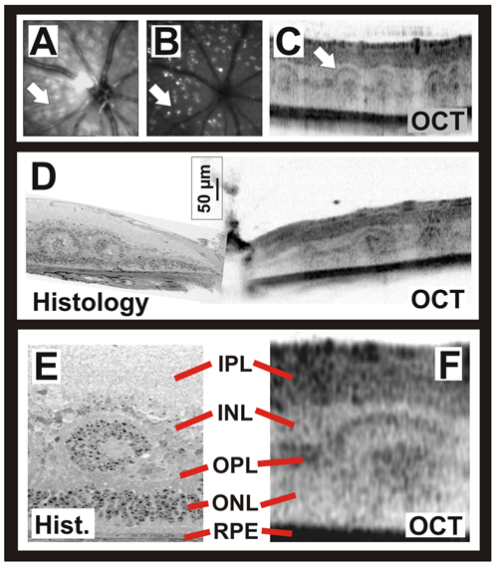
Capability of the OCT to detect and capture the nature of lesions. A)–F) Multiple retinal rosette formation in the neural retinal leucine zipper (*Nrl*) knockout mouse. A) SLO surface image (514 nm) in which the retinal rosettes (arrow) show as whitish dots. B) SLO autofluorescence image indicating that the rosettes contain fluorescent material. C) Representative OCT slice of a *Nrl* knockout mouse revealing details of the nature of the rosettes (arrow) and their depth localization. D) Comparison of the OCT representation of *Nrl* rosettes with histology (different individual animal). E, F) Detail illustrating how well retinal structures in OCT and histology correlate.

In the *Nrl* knockout mouse, the neural retina leucine zipper (*Nrl*) gene is impaired [8]. A major function of Nrl is to determine the fate of rod photoreceptor precursor cells, so that a lack of Nrl during development leads to an abnormal differentiation of these rod precursors into cone-like photoreceptors. The corresponding human disease, enhanced S-cone syndrome (ESCS), may either be caused by a lack of NRL or NR2E3, a transcription factor with overlapping functions to NRL [9]. A landmark of such retinas is the formation of rosettes [9], [10], whose distribution can be assessed with conventional *in vivo* imaging as they present as whitish dots in native SLO (Fig. 4 A) and in autofluorescence mode (Fig. 4 B). The structure of the respective lesions becomes accessible with OCT both in humans [9] and mice (Fig. 4 C). As before, histomorphological and OCT data correlate well (Fig. 4 D). The detailed comparison (Fig. 4 E vs. Fig. 4 F) illustrates the difference in image appearance between reflection- and absorption-based methods discussed before (Fig. 1).

### *Crb1* knockout mouse

**Figure 5:**
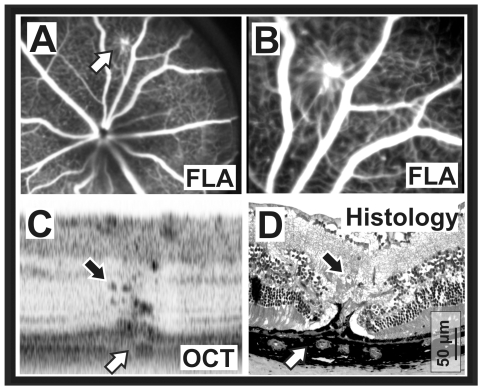
Capability of the OCT to detect and capture the nature of lesions. A)–D) Site of neovascularization in a Crumbs 1 (*Crb1*) knockout mouse. A) SLO fluorescence angiographic (FLA) image of a retinal neovascular site (arrow) in a representative *Crb1* knockout mouse. B) Detail of g) illustrating the traction the aberrant vessel applies to the neighboring capillaries. C) Representative OCT slice depicting enlarged aberrant retinal vessels (black arrow) as well as choroidal vascular changes (white arrow) at the same position, implicating a connection between both vascular beds. D) Histological section of the above neovascular site for comparison. The black arrow points to aberrant retinal vessels, and the white arrow to choroidal changes.

It is almost impossible to detect scattered lesions functionally, particularly in cases of vascular abnormalities without generalized hypoxia. In mutants lacking the Crumbs1 gene, the separation between inner and outer retina is weakened [11], allowing unnatural migration of cells and cell groups across gaps within that border [12]. It is believed that if a capillary vessel runs close to such a site, it may also descend into the vessel-free photoreceptor layer and eventually make contact with the retinal pigment epithelium (RPE). As the RPE produces VEGF, such aberrant vessels usually proliferate and appear in some cases to form connections with the choroidal vascular system. The sheer presence of such neovascularizations may be detected with traditional fluorescein angiography (FLA, Fig. 5 A, B). However, an accurate topographic reconstruction that allows to uncover the exact depth localization and the extent of the lesion is now feasible with the OCT (Fig. 5 C). This particular example further illustrates the capability to detect intraretinal (black arrows) as well as choroidal vessels (white arrows), which so far required a full histological work-up of such lesion sites (Fig. 3D).

### *Rb* retina-specific knockout

**Figure 6:**
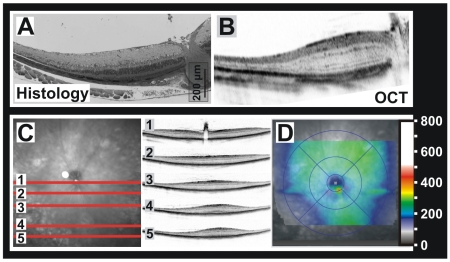
Topographic analysis of retinal thickness in an organ-specific model of retinoblastoma protein (*Rb*) deficiency. Thickness variations (center vs. periphery) were caused by imperfections of the Cre-lox system (see text), leading to differences in developmental apoptosis. A) Histological section across the central retina showing the smooth transition between centrally normal and peripherally reduced thickness. B) OCT section of the same region, the retinal thickness correlating well with the histomorphological data. C) Assessment of the gradual changes of retinal thickness from center to (mid)periphery based on 5 manually placed OCT slices. Left: SLO image of the fundus region with the position of the slices superimposed. Right: OCT slices at the positions indicated, ordered from center to periphery. D) Topography of retinal thickness calculated from 92 equidistant OCT slices (“volume scan” data). The color scale values are in µm.

Interference with the molecular patterning events during retinal development often results in cell-type specific dysmorphogenic alterations. An example is the retinoblastoma protein (Rb), an important tumor suppressor, that blocks cell division and death by inhibiting the E2f transcription factor family, leading to apoptosis of a large fraction of retinal cells [13]. As factors like Rb often have related functions in many different tissues, a complete loss of respective genes commonly leads to embryonic lethality, preventing the generation of knock-out models. However, organ-specific models are often possible to obtain on the basis of the Cre-*lox* P system, where the gene to be deleted is solely removed in tissues that express Cre recombinase [14]. In this work, we used *Rb^loxP/loxP^;α-Cre* mice where the floxed *Rb* exon 19 is only deleted in the retina at embryonic day E10 [15]. However, the expression of the *α-Cre* transgene is not topographically homogeneous in this mouse model [16], leading to different degrees of gene inactivation within the same organ. In particular, the retinal periphery shows the full effect of Rb loss, whereas there is almost no *α-Cre* transgene activity in the central retina. Here, we took advantage of this Cre recombinase distribution pattern to ascertain the transition effect between the central area with regular development and more peripheral parts with developmental apoptosis within the same retinal slice (Fig. 6). Despite the somewhat lower resolution achievable in living tissues, the changes in retinal layering and thickness associated with *Rb* gene loss appeared equally well ascertainable with histology and OCT (Figure 6 A vs. B). An important feature of OCT is the capability to capture multiple (Fig. 6 C) sections directly linked to the surface image within a short period of time, allowing to follow topographical changes like the thickness gradient from center to periphery. A set of consecutive serial sections (“volume scan”) even allows this gradient to be mapped (Fig. 6 D), which in this case correlates with the topography of *α-Cre* transgene expression *in vivo*.

## Discussion

In this work, we assessed the scientific benefit of the OCT in rodent models featuring retinal neurodegeneration, perturbed retinal structure, neovascular conditions, and developmental aberrations, all likely fields of interest to a wide range of academic and industrial researchers.

The results in the light damaged retina show the benefit of the combination of surface imaging with the SLO for aiming at sites of interest (like here demarcated by autofluorescence) and the OCT. We found a close correlation of the site of autofluorescence in the 488 nm SLO images (attributed to the lipid-rich remnants of the photoreceptor outer segments) with the outer retinal damage in the OCT sections. For the first time, we were able to demonstrate edema formation in these rodents (Fig. 3 E), which appears to be part of the dynamic processes associated with acute light exposure and, because of the tissue processing, is not detectable in conventional morphological analyses.

An example of perturbed retinal structure is the rosette formation in the *Nrl* knockout mouse (Fig. 4). Whereas the relatively even topographical distribution of the lesions became visible in SLO imaging (similar to a retinal whole-mount, Fig. 4 A,B), details of the structure were accessible *in vivo* with OCT sections (Fig. 4 E,F). The formation of rosettes in Nrl mutants is not yet fully understood, but it is known that cones are a major component of these dysmorphic structures, with their inner and outer segments pointing towards the center of each rosette [10]. The respective photoreceptor nuclei around the center of the rosette in Fig. 4E (similar to the ONL ones) correspond in the OCT to an area of low reflectivity in Fig. 4F (similar to the ONL band). The center of rosettes was further shown to contain RPE proteins and have connections to the RPE layer [10]. In the OCT, we found that this tissue has reflective properties close to plexiform layers (Fig. 4F). Rosette formation occurs postnatally [10], [17], apparently driven by a metabolic need of the retina, leading to an increased surface area between RPE and photoreceptors. To better understand this process, we will examine the time course in individual animals in a separate study.

In mutants lacking the *Crumbs1* gene, lesions are commonly sparse (Fig. 5 A), which makes an histological analysis laborious and demanding. An *in vivo* screening with the SLO has proven to be very effective the overall assessment as well as a preselection for immunostainings, saving time, money, and animals. Now, with the OCT, the nature and extent of the lesions can be assessed in real time. The particular example in Fig 5 C illustrates the capability to detect intraretinal (black arrows) as well as choroidal vessels (white arrows), which so far required a full histological work-up of such lesion sites (Fig. 5 D). Having the morphological information available *in vivo* permits to study the dynamics of such sites in more detail (together with the use of dyes) before the animal is sacrificed.

The results in the *Rb^loxP/loxP^;α-Cre* mice illustrate how the developmental apoptosis due to an imbalance of RB and the E2Fs [13], [15] affects the different layers (Fig. 6).

However, developmental apoptosis only occurs in areas where *Rb* is missing, which in this conditional knock-out is the area of Cre expression [14]. Since the retina-specific *α-Cre* mice used here do show a central-peripheral expression gradient, so does the apoptosis and, subsequently, retinal thickness. So, besides the *Rb*-specific findings, this model also helped to assess the feasibility of a topographical mapping of retinal thickness based on a set of consecutive serial sections (“volume scan”). The color-coded map (Fig. 6 D) reveals regular retinal thickness values mainly around the optic disc (image center), which reflects the inverse topography of the *α-Cre* transgene expression [16]. Thickness maps are further useful to follow thickness changes over time.

The prime advantage of OCT is certainly the ability to non-invasively produce histology-analogue retinal sections. As we have shown, these are both useful for the assessment and numerical quantification of generalized changes and for the detection and analysis of sporadic, localized lesions.

In many cases, standard histology may be replaced or at least its extent reduced by OCT, diminishing the amount of study animals needed. Even if histological sections are required (e.g. immunostains), OCT may help to preselect and to determine the optimal time point in individual animals in case of progressive changes.

As most degenerative processes are dynamic in nature, the non-invasive examination has substantial advantages for the understanding of short–term alterations like edema formation. A requirement for preclinical and long-term studies is that markers of potential degenerative changes or therapeutic effects may be analyzed numerically. The OCT, for the first time, allows an accurate quantitative description of the retinal layers *in vivo* as shown in Fig. 2. As the majority of lesions primarily compromise the outer retina but leave the inner retina unchanged, it is a fundamental improvement that these layers are now accessible to separate quantification.

In summary, our results present the OCT as an important new tool for the *in vivo* analysis of rodent eyes. It is not only a methodological step forward but will also significantly help to reduce standard histology and thus the amount of animals needed. The ability to monitor developmental as well as inherited and induced degenerative processes and respective therapeutic intervention in the same individual animals opens a wide field of applicability in future long-term and preclinical studies. In conjunction with other non-invasive tests like electroretinography (ERG), OCT allows for a refined morphological and functional follow-up over time.

## Methods

### Animals

All procedures concerning animals adhered to the ARVO statement for the use of animals in ophthalmic and vision research, and were performed with permission of local authorities (Regierungspraesidium Tuebingen). All mice were kept under a 12 h∶12 h light-dark cycle (60 lux), had free access to food and water and were used irrespective of gender.

### Optical adaptation to the mouse eye

Retinal imaging of the mouse eye with its small pupil has been a challenge because of the difficult alignment of light delivered to the eye. The small aperture also reduces the amount of light which is reflected from the retina, and therefore decreases the signal-to-noise ratio [18]. Over a decade of SLO in vivo imaging in animals, we have tested several conditions for the visualization of the murine fundus. We found that a basic setup containing only two additional lenses is sufficient to obtain very good quality results without the need to modify the equipment internally (Figure 1D). All SLO and OCT images in this work were recorded using this setup. On the SLO/OCT side, a 78 diopter double aspheric lens (Volk Optical, Inc., Mentor, OH 44060, U.S.A.) is fixed directly to the outlet of the device. To the eye of the mouse, a custom-made contact lens with a focal length of 10 mm is applied with a drop of methyl cellulose (Methocel 2%, OmniVision, Puchheim, Germany). Mouse eye and equipment must be aligned correctly, e.g. by means of a XYZ table, and brought closely together, so that they almost touch.

### Scanning-laser ophthalmoscopy and angiography

For en face retinal imaging, we used the commercially available HRA 1 and HRA 2 systems (Heidelberg Engineering, Dossenheim, Germany) featuring up to two Argon wavelengths (488 and 514 nm) in the short wavelength range and two infrared diode lasers (HRA 1: 795 and 830 nm, HRA 2: 785 and 815 nm) in the long wavelength range. The 488 and 795 nm lasers are used for fluorescein (FLA) and indocyanine green (ICG) angiography, respectively. Appropriate barrier filters at 500 and 800 nm remove the reflected light with unchanged wavelength while allowing only the light emitted by the dye upon stimulation to pass through.^1^

A detailed protocol for anesthesia and conventional imaging is described elsewhere [1]. Briefly, mice were anaesthetized by subcutaneous injection of ketamine (66.7 mg/kg) and xylazine (11.7 mg/kg) and their pupils dilated with tropicamide eye drops (Mydriaticum Stulln, Pharma Stulln, Stulln, Germany) before image acquisition.

### Spectral domain Optical Coherence Tomography

SD-OCT imaging was done in the same session as cSLO to minimize variability. For OCT imaging, we used a commercially available Spectralis™ HRA+OCT device from Heidelberg Engineering featuring a broadband superluminescent diode at λ = 870 nm as low coherent light source.

Each two-dimensional B-Scan, recorded with the equipment set to 30° field of view, consists of 1536×496 pixels, which are acquired at a speed of 40,000 scans per second. Optical depth resolution is ca. 7 µm with digital resolution reaching 3.5 µm [19]. The combination of scanning laser retinal imaging and SD-OCT allows for real-time tracking of eye movements and real-time averaging of OCT scans, reducing speckle noise in the OCT images considerably. Resulting data were exported as 24 bit color bitmap files and processed in Adobe Photoshop CS2 (Adobe Systems, San Jose, CA, U.S.A.).

### Analysis of retinal morphology

Animals were sacrificed and enucleated for histological analysis. After orientation was marked, the eyes were fixed overnight in 2.5% gluteraldehyde prepared in 0.1 M cacodylate buffer and processed as prescribed previously [20]. Semi-thin sections (0.5 mm) of Epon-embedded tissue were prepared form the central retina, counterstained with methylene blue and analyzed using a light microscope (Axiovision, Zeiss, Jena, Germany).
